# The Triple-Double Technique of Arthroscopic Hill-Sachs Remplissage

**DOI:** 10.1016/j.eats.2023.04.024

**Published:** 2023-08-07

**Authors:** Adam J. McNulty, Robert U. Hartzler

**Affiliations:** a Department of Orthopaedic Surgery, UT Health San Antonio, San Antonio, Texas, U.S.A.; b Burkhart Research Institute for Orthopaedics, TSAOG Orthopaedics and Spine, San Antonio, Texas, U.S.A.

## Abstract

Surgeons are increasingly treating Hill-Sachs lesions arthroscopically by suturing the posterior rotator cuff and capsule into the defect, a procedure known as “remplissage.” A number of remplissage techniques have been described in the literature, and these often vary by the number and location of suture anchors. The “triple-double” technique of arthroscopic Hill-Sachs remplissage can be used for larger lesions. This technique utilizes a three-anchor configuration secured by interconnected double-mattress sutures to provide durable fixation through a large contact area between the capsulotenodesis tissue and the prepared bone bed to theoretically optimize healing.

## Introduction

The Hill-Sachs lesion was first defined in 1940 as a “compression fracture of the posterolateral aspect of the articular surface of the humeral head, as it impinges on the anterior rim of the glenoid fossa”.^1^ In 2000, Burkhart and De Beer demonstrated that in the presence of either an engaging Hill-Sachs lesion or glenoid bone loss resulting in an “inverted pear” configuration, isolated Bankart repair is associated with a high rate of failure and recurrent instability.^2^ The concept of the glenoid track in the setting of bipolar bony lesions was then described in 2014 by Di Giacomo et al. In the case of an “off-track” lesion, the Hill-Sachs lesion is theorized to engage the anterior glenoid rim and an isolated anterior soft tissue procedure is more likely to fail.^3^ Surgeons are increasingly addressing the Hill-Sachs lesion as a way to reduce recurrent anterior instability after isolated arthroscopic Bankart repair, and rarely to address persistent engagement or instability after a glenoid bony reconstruction (e.g., Latarjet).

The arthroscopic “remplissage” technique was originally described in 2008 as an arthroscopic tenodesis of the posterior rotator cuff and capsule to fill the Hill-Sachs lesion and increase both active and passive stability.^4^ This procedure has resulted in good postoperative clinical outcome scores, minimal loss of shoulder motion, high rates of short-term healing, and an overall low complication rate.^5^ A recent meta-analysis has demonstrated that Bankart repair with remplissage resulted in fewer cases of recurrent instability when compared to isolated Bankart repair in patients with Hill-Sachs lesions and subcritical glenoid bone loss.^6^ Traditionally, arthroscopic remplissage has been performed with one or two suture anchors. Occasionally, the Hill-Sachs lesion is large enough to add additional anchors. In the current technique, the authors describe a remplissage technique using a three-anchor configuration secured by interconnected double-mattress sutures.

## Surgical Technique

**Fig 1:**
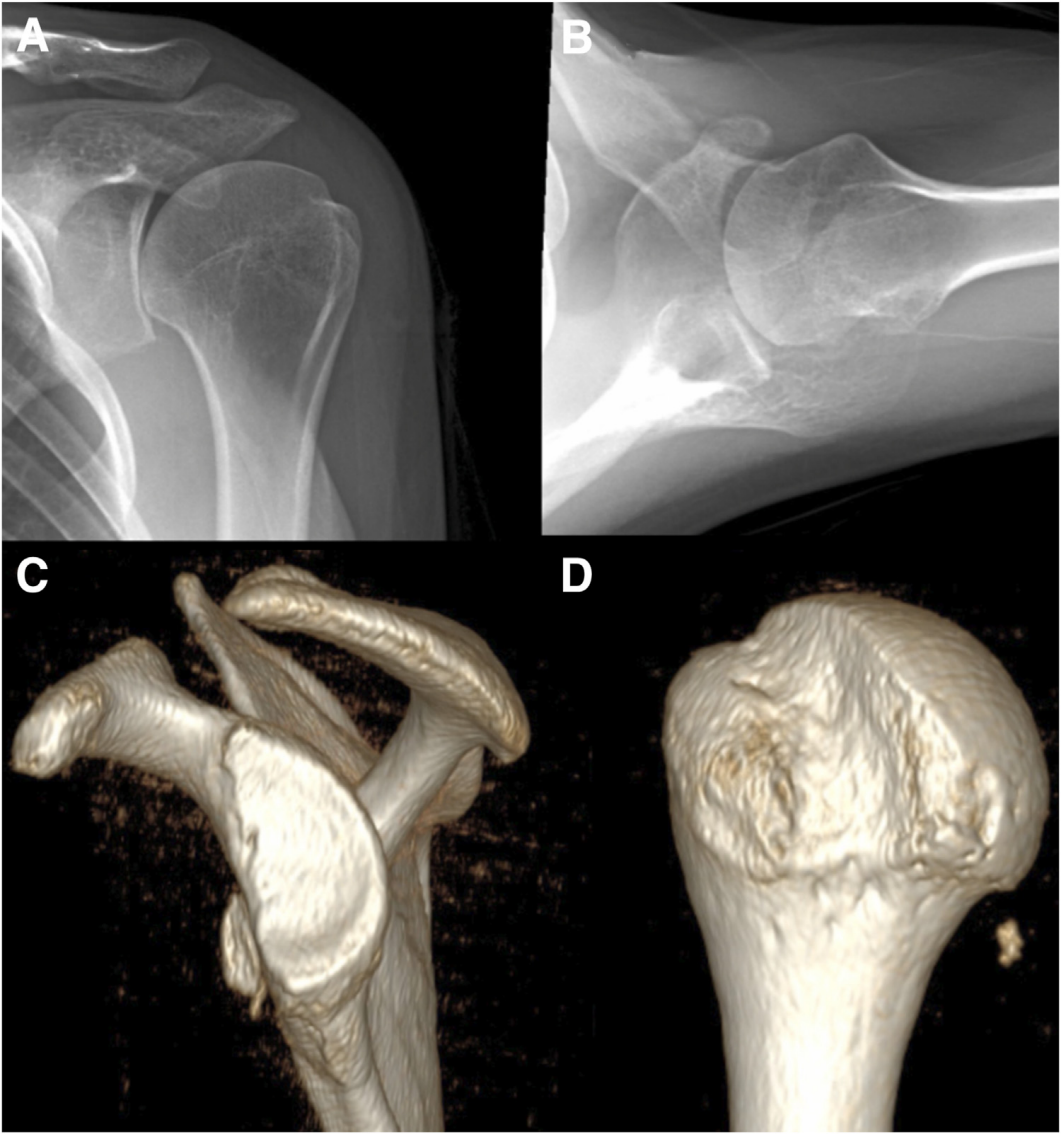
Preoperative radiographs and three-dimensional (3D) computed tomography (CT) reconstruction of the left shoulder. The large Hill-Sachs defect, as well as the notable glenoid bone loss and anterior subluxation of the humeral head can be appreciated. (A) Left shoulder anterior-posterior radiograph. (B) Left shoulder axillary lateral radiograph. (C) Left glenoid 3D CT reconstruction showing magnitude of glenoid bone loss. (D) Left humeral head 3D CT reconstruction showing size of Hill-Sachs lesion.

The authors’ preferred position for shoulder arthroscopy is lateral decubitus. If the remplissage is done in conjunction with an open procedure such as a Latarjet procedure, the open portion may first be done in the beach chair position. If the Hill-Sachs lesion is still found to engage the anterior glenoid rim on examination of the shoulder after addressing the glenoid bone loss (Fig 1), the patient may be repositioned in the lateral decubitus position for arthroscopic remplissage.

The procedure begins with creation of a standard posterior viewing portal and diagnostic arthroscopy, followed by creation of a cannulated anterosuperior portal just off of the anterolateral corner of the acromion (Table 1). If done in conjunction with arthroscopic Bankart repair, the surgeon may also create additional portals for standard arthroscopic Bankart repair techniques at this time. Following standard diagnostic arthroscopy, the camera is switched to the anterosuperior portal, and the posterior portal is cannulated to allow for easier passing of instruments and sutures. The posterior capsule and synovium are debrided with an arthroscopic shaver.

**Table 1:** Surgical Steps

Surgical Steps	
1. Diagnostic arthroscopy	Begin the procedure with a standard diagnostic arthroscopy using a posterior viewing portal. Introduce and cannulate an anterosuperior portal just off the anterolateral border of the acromion.
2. Evaluation and preparation of Hill-Sachs lesion	Using the anterosuperior portal for viewing, switch to a 70° arthroscope and evaluate the Hill-Sachs lesion and cannulate the posterior portal. Use an arthroscopic shaver and ring curette or rasp to prepare the bone bed. The anterosuperior viewing portal and the 70° arthroscope are also used for steps 3-5.
3. Placement of two medial anchors	Introduce two medial anchors through the posterior cannulated portal. These should be located at the medial margin of the Hill-Sachs lesion. Place the inferior anchor followed by the superior anchor.
4. Percutaneous shuttling of medial sutures	Introduce a spinal needle percutaneously through the capsule and rotator cuff at the appropriate location for the capsulotenodesis. Pass a 0 or #1 PDS suture through the spinal needle into the joint. Use a grasper to pull the free end of the PDS suture through the posterior cannulated portal. Exchange this for a looped shuttling suture and then shuttle all four suture strands through the rotator cuff and capsule. Do this first for the superomedial anchor and then for the inferomedial anchor.
5. Percutaneous placement of lateral anchor	Pass spinal needle percutaneously at the appropriate location, and then replace it with the drill bit for the anchor. Pass drill guide over the drill bit and down to bone, and then drill and place anchor.
6. Subacromial debridement and bursectomy	Using a 30° arthroscope and the posterior portal for viewing, create a standard lateral portal. Carefully begin a subacromial bursectomy and debridement, and then switch to the lateral portal for viewing. Be cautious with the shaver, as it can damage the sutures. Electrocautery is safer to use in close proximity to the sutures. Once the bursectomy and debridement are complete, the surgeon should be able to visualize the three sets of sutures passing through the space. Recannulate the posterior portal in the subacromial space.
7. Tying of double mattress sutures	Retrieve one suture limb from two of the anchors through the posterior cannulated portal and tie outside of the body. Retrieve the other 2 ends of those suture limbs, and tension them to bring the first knot down to the rotator cuff/capsule tissue, and then tie a static arthroscopic knot to create the double mattress. Repeat two more times to interconnect all 3 anchors.
8. Evaluate repair	Insert 70° camera back into the glenohumeral joint from the lateral portal to inspect the capsulotenodesis. The Hill-Sachs defect should now be filled.

**Fig 2:**
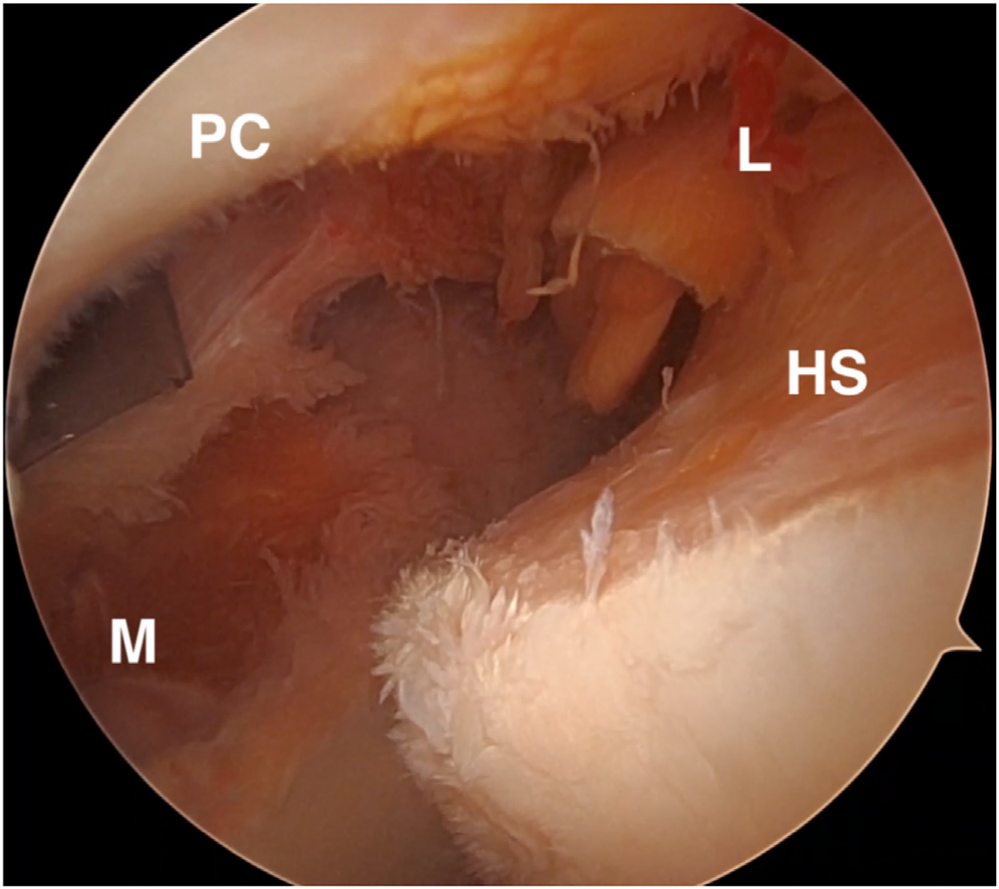
Arthroscopic image of the left shoulder, demonstrating a Hill-Sachs lesion viewed from the anterosuperior portal with a 70° arthroscope. Once diagnostic arthroscopy is completed, and a cannulated anterosuperior portal is introduced, the arthroscope is moved to the anterosuperior portal, and a 70° scope is used. HS, Hill-Sachs lesion; PC, posterior capsule; L, lateral; M, medial.

The authors liberally change between 30° and 70° arthroscopes as needed to best visualize the lesion and perform the repair steps. The Hill-Sachs lesion is inspected, and the size of the lesion and its position relative to the articular surface and posterior insertion of the rotator cuff should be evaluated (Fig 2). Using the posterior portal as the working portal, the defect is then prepared with a ringed curette and an arthroscopic shaver to achieve a healthy, bleeding bone bed for improved tissue healing (Video 1).

**Fig 3:**
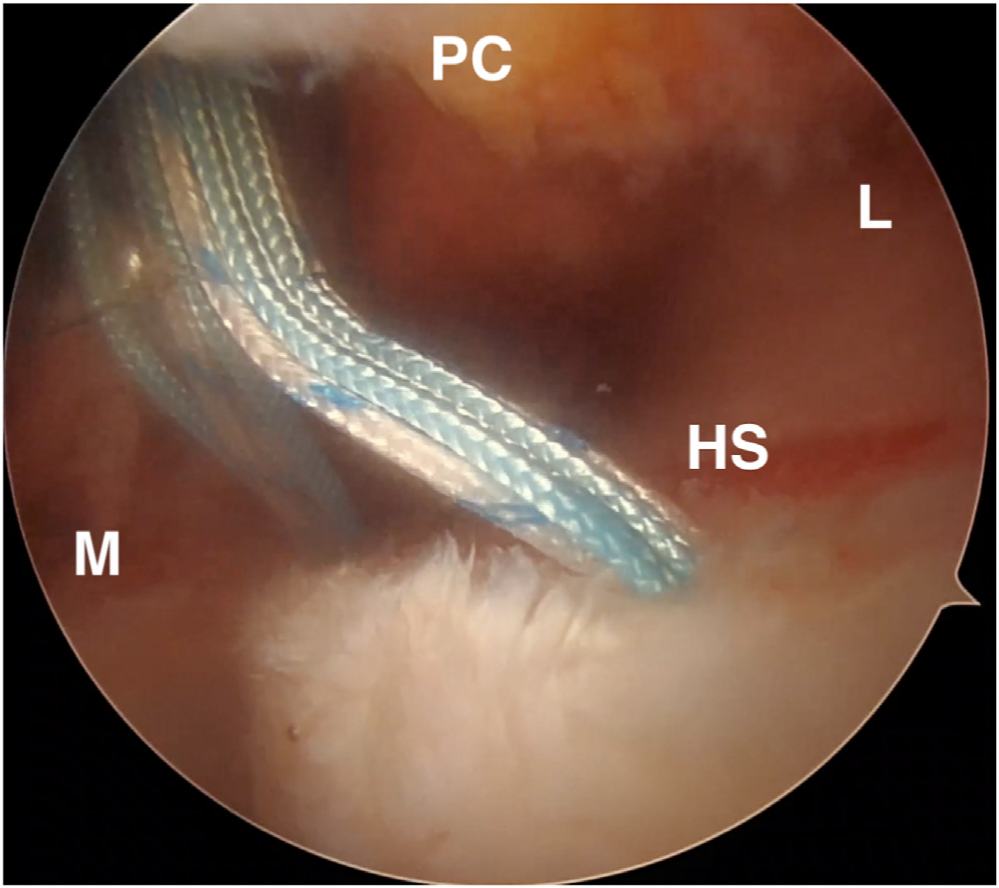
Placement of the superomedial and inferomedial anchors. Viewing the left shoulder from the anterosuperior portal with a 70° camera, and the bone bed is prepared using an arthroscopic shaver and a rasp. The two medial anchors are then placed through the posterior cannulated portal. Once both medial anchors are placed, the sutures are passed through the rotator cuff and the capsular tissue using the percutaneous shuttling technique described above. HS, Hill-Sachs lesion; L, lateral; M, medial; PC, posterior capsule.

Once the bone is prepared, the two medial anchors are placed through the posterior cannulated portal (Fig 3). The 2.3-mm all-suture inferomedial anchor (Iconix; Stryker, Greenwood Village, CO) is placed first followed by the superomedial anchor, and these should be located at the most medial aspect of the Hill-Sachs defect. The sutures are passed through the rotator cuff and capsule using a percutaneous shuttling technique using a spinal needle advanced through the capsule and rotator cuff further laterally than the posterior cannulated portal (Video 1). PDS suture (0 or #1) is passed through the spinal needle into the joint and using a grasper, the free end of the PDS suture is then pulled back out of the joint through the posterior portal. The PDS is then exchanged for a looped shuttling suture such that the looped end of the shuttling suture is exiting through the posterior cannula, while the free end is exiting percutaneously (Video 1). This looped shuttling suture is used to pull all four suture strands through the rotator cuff and capsule, beginning with the superomedial sutures. The shuttling steps are repeated for the inferomedial sutures.

**Fig 4:**
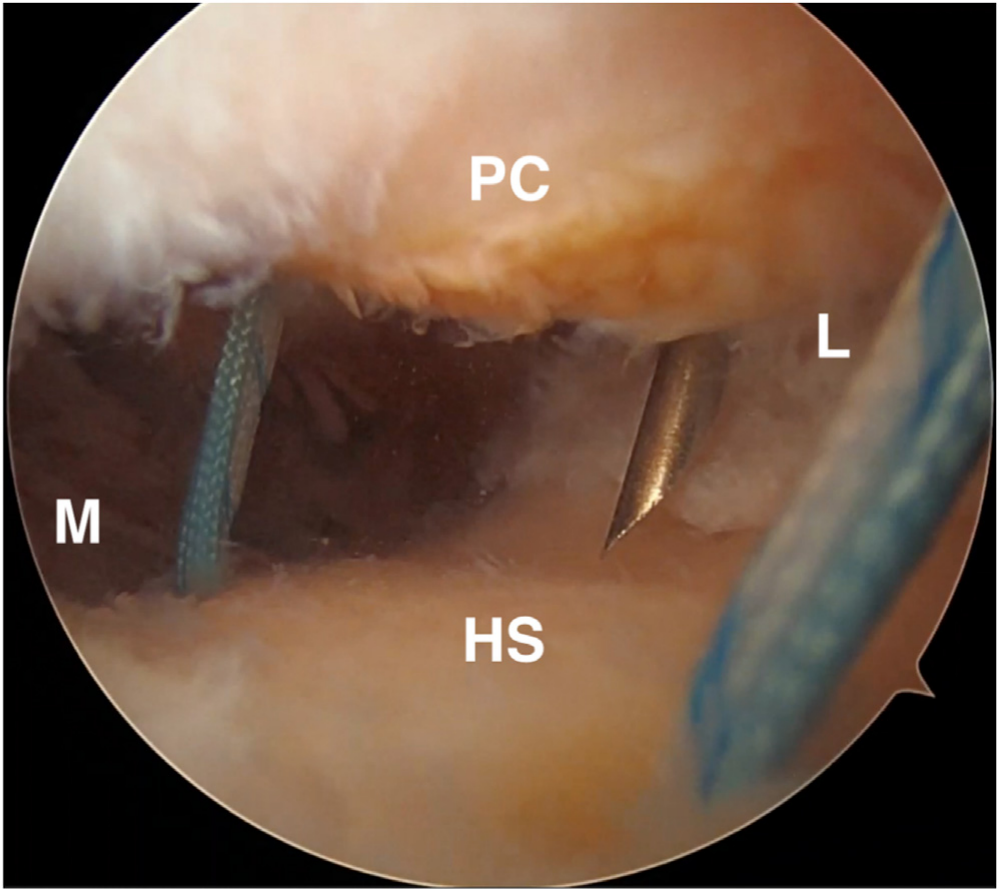
The lateral anchor is placed percutaneously. A spinal needle is inserted to achieve the proper placement. This is then replaced by the drill bit used for the anchor placement. Once the drill bit is in place, the guide sleeve is slid over the drill bit and down to the bone and the lateral anchor is placed. HS, Hill-Sachs lesion; L, lateral; M, medial; PC, posterior capsule.

**Fig 5:**
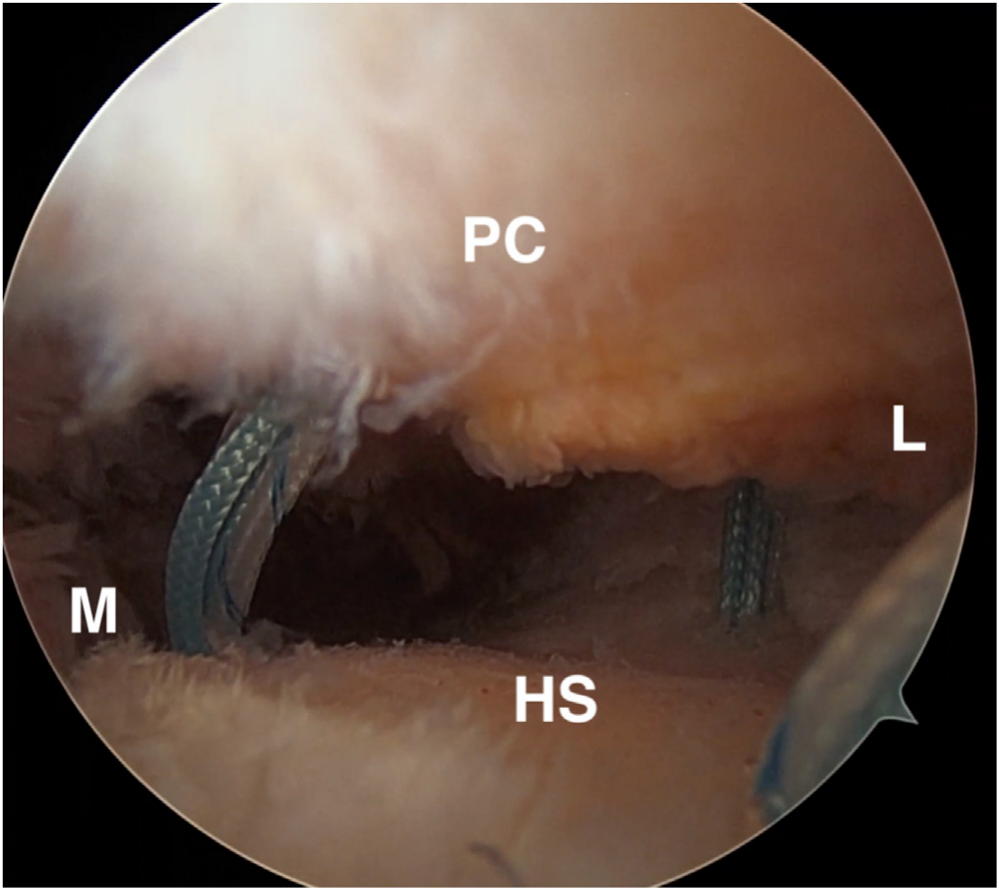
Final view of the three anchors placed in a triangular configuration with the sutures passed through the posterior rotator cuff and capsular tissue, viewed through the anterosuperior portal using a 70° arthroscope. HS, Hill-Sachs lesion; L, lateral; M, medial; PC, posterior capsule.

The lateral anchor is then placed in a transtendinous fashion. The authors start by placing a spinal needle percutaneously at our desired anchor location. This should be at the far lateral margin of the Hill-Sachs lesion (Fig 4) and centered at a height between the two medial anchors. Next, the spinal needle is exchanged by a drill bit placed transtendinously. Once the drill bit is in place, the guide sleeve is slid over the drill bit and down to the bone, and the lateral anchor is drilled and placed (Fig 5). At this point, the three anchors (superomedial, inferomedial, and lateral) should create a triangular configuration with the four suture strands from each respective anchor passing through the rotator cuff and capsule at their appropriate locations.

**Fig 6:**
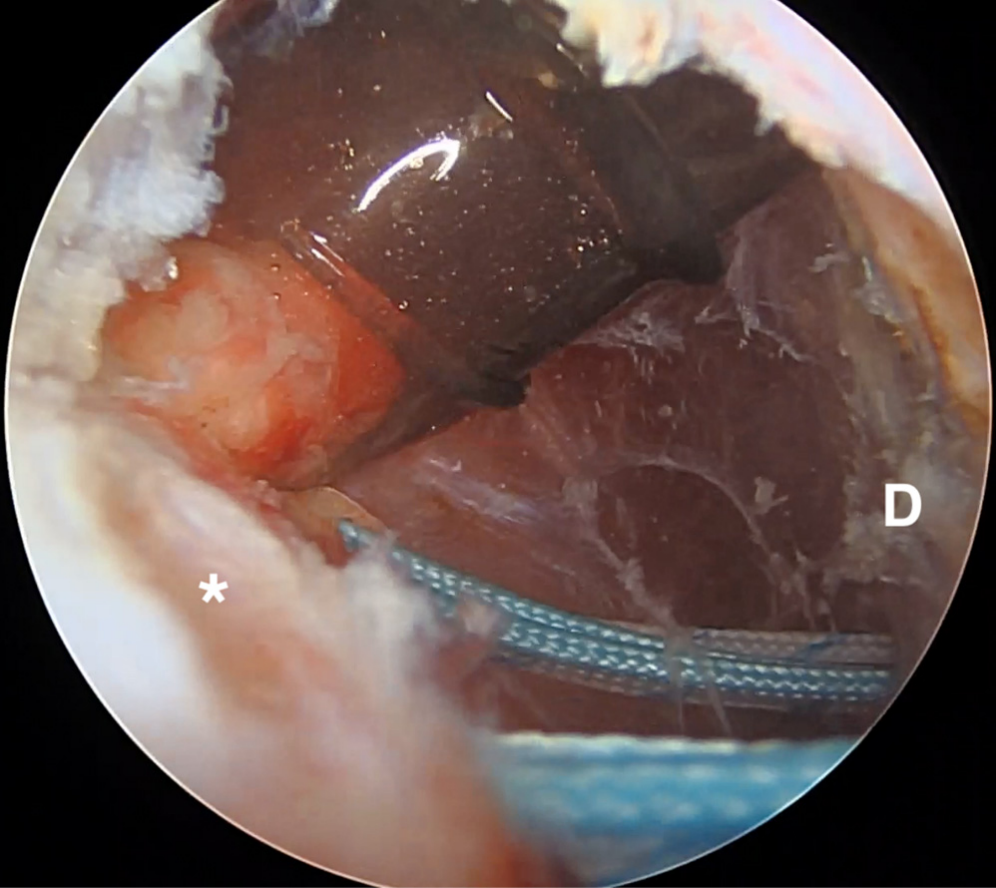
Extra-articular view from the lateral portal with the patient in a lateral decubitus position after subacromial debridement and bursectomy. The three pairs of sutures are visualized passing through the rotator cuff and capsule. The bursectomy is performed primarily with electrocautery in order to avoid damaging the sutures with an arthroscopic shaver. The posterior portal is cannulated for knot tying. D, deltoid. The asterisk (∗) denotes posterior capsulotenodesis tissue.

**Fig 7:**
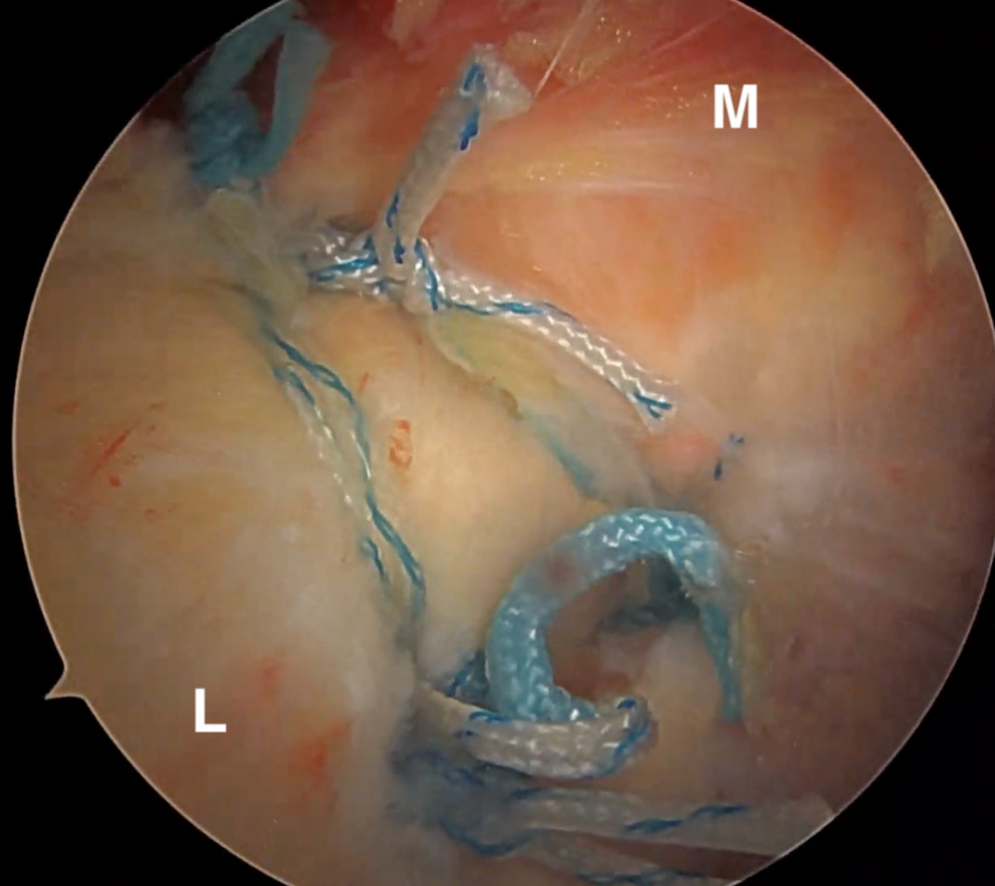
Final extra-articular view from the lateral portal showing the triple-double configuration of the three anchors with interconnected double-mattress sutures. The capsule and rotator cuff tissue has been well reduced into the Hill-Sachs defect. L, lateral; M, medial.

**Fig 8:**
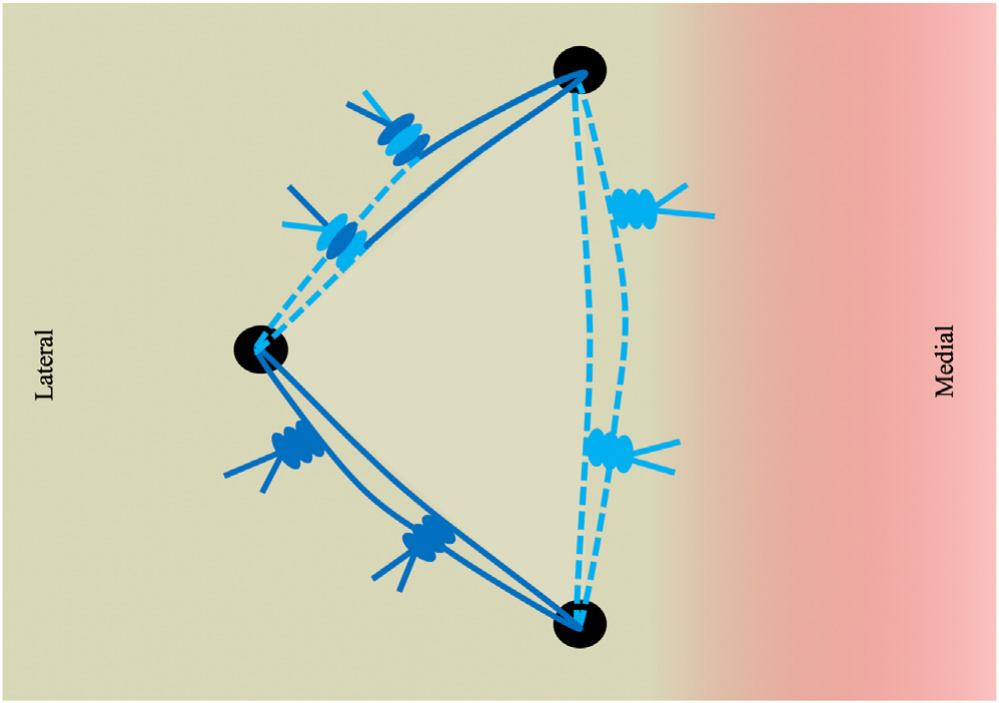
Depiction of the “triple-double” configuration of three double-loaded suture anchors interconnected by double-mattress sutures.

**Fig 9:**
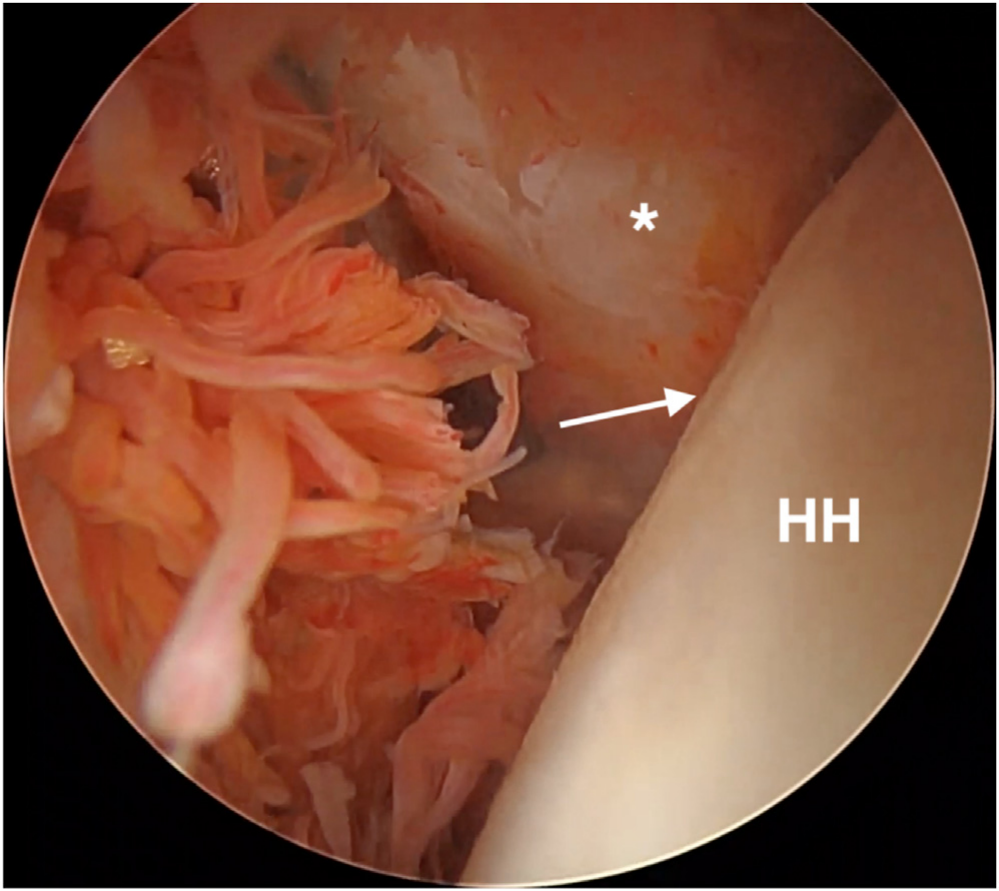
Final intra-articular view from the anterosuperior portal showing the capsular and rotator cuff tissue filling the void of the Hill-Sachs defect. The defect is no longer visible from an intra-articular viewpoint (arrow). HH, humeral head. The asterisk (∗) denotes posterior capsulotenodesis tissue.

The camera is then moved to the posterior portal and a standard lateral portal is introduced. Subacromial debridement and bursectomy is performed, taking care not to damage the sutures passing through the posterior capsule and rotator cuff. The authors then switch to the lateral viewing portal with the 30° scope and use electrocautery to further debride this space posteriorly (Fig 6). Once a clear view of the three suture pairs has been obtained, begin the process of tying interconnected double mattresses between anchors by retrieving one suture limb from the first two anchors through the posterior cannula. These are tied together outside of the body. The authors then retrieve the other ends of those two sutures through the posterior cannula. Pulling tension on these two ends will bring the previously tied loop down over the tissues. These two free ends are then tied arthroscopically with 6 half hitches to create a static knot. This process is repeated 2 more times to create a total of 3 interconnected double mattresses between the 3 anchors (Figs 7 and 8). The camera is then placed back into the glenohumeral joint through the anterosuperior portal to confirm that the rotator cuff and capsular tissue has been inset into the Hill-Sachs defect (Fig 9).

## Discussion

Various surgical procedures and techniques have been described with regard to treating anterior shoulder instability. Factors such as the amount of glenoid bone loss and the presence, size, and geometry of a Hill-Sachs lesion are the major determinants when it comes to devising a treatment plan (Table 2).

**Table 2:** Pearls and Pitfalls

Pearls	Pitfalls
Ensure anchors are at medial and lateral margins of the Hill-Sac**h**s lesion to allow for maximum contact area for capsulomyotenodesis.	There is often a notable learning curve when viewing from the anterosuperior portal while using a 70° arthroscope.
Use percutaneous PDS shuttling technique to pass sutures from medial anchors through rotator cuff and capsular tissue at appropriate positions.	Pass all 4 suture strands from each respective anchor together to avoid tissue bridges.
Perform thorough debridement of subdeltoid bursa to achieve good visualization when tying knots, and confirm adequate reduction of tissues into the defect.	Overaggressive subdeltoid bursectomy with an arthroscopic shaver may result in damage to the sutures if care is not taken.
Tie the first knot for each double mattress suture configuration outside of the body to improve efficiency and reduce surgical time. The second knot in the double mattress cannot be a sliding arthroscopic knot**—**it must be static (e.g.**,** alternating half-hitches).	Once the anchors are interconnected, visualization for anterior soft tissue work will be more difficult. Complete this prior to tying the remplissage knots.

In considering treatment of a Hill-Sachs lesion, the concept of the “glenoid track” currently plays a large role in the authors’ surgical decision-making. The glenoid track is defined as the zone of contact between the glenoid and the humeral head as the shoulder goes through a range of motion.^3^ In the presence of anterior glenoid bone loss, a Hill-Sachs lesion may either be “on-track” or “off-track”. An “on-track” lesion will not engage the anterior glenoid rim, while an “off-track” lesion may engage the anterior glenoid rim, resulting in recurrent instability. There are 2 ways to address an “off-track” lesion—either by increasing the size of the glenoid track with a Latarjet or other bone grafting procedure, or by decreasing the size of the Hill-Sachs lesion. In severe circumstances, both of these may be indicated, such as in the current case.

When it comes to addressing a Hill-Sachs lesion, the size of the lesion can help to guide decision making. In a review article by Provencher et al., lesions involving <20% of the humeral head articular surface were rarely found to be clinically significant, while lesions of >40% of the articular surface were nearly always clinically significant and often responsible for recurrent instability.^7^ These clinically significant lesions may be treated with humeral head bone augmentation (tricortical iliac graft, humeral head allograft, femoral head allograft), prosthetic resurfacing, or a remplissage procedure. Biomechanically, remplissage has shown favorable results when it comes to restoring glenohumeral stability in bipolar bone loss, as shown by Hartzler.^8^ A systematic review published in 2019 concluded that the addition of remplissage to a Bankart repair resulted in lower recurrence rates and better shoulder function when compared to an isolated Bankart repair, while also largely preserving shoulder external rotation range of motion.^9^ Latarjet is also effective at reducing recurrent instability; however, Yang et al. have shown a significantly higher complication rate with Latarjet procedure compared to Bankart repair with remplissage.^10^

For large Hill-Sachs lesions, the described technique for arthroscopic remplissage provides robust capsulotenodesis fixation to fill the Hill-Sachs defect, while providing direct visualization during knot tying. By using a three-anchor configuration secured by interconnected double-mattress sutures, the triple-double technique theoretically allows compression of a large contact area between the capsular tissue and the bony defect to optimize healing. Consigliere et al. has previously described a three-anchor technique for Remplissage with all three anchors being interconnected.^11^ The authors prefer the triple-double technique since a single point of suture breakage or knot unraveling in the pulley system will not result in complete loss of fixation of the remplissage. One of the risks associated with this technique involves the subdeltoid bursectomy step. Care must be taken not to damage the sutures when debriding the subdeltoid bursa, as the sutures are in close proximity. A limitation of the triple-double technique is the technical difficulty of the procedure. The author prefers to use a 70° arthroscope for the majority of the cases to achieve better visualization of the defect, and there may be a steep learning curve for a surgeon who does not routinely use a 70° arthroscope.

In conclusion, the triple-double technique of arthroscopic remplissage described here employs three anchors in a triangular configuration secured by interconnected double-mattress sutures to provide a large contact area to promote healing.

## Supplementary Data

ICMJE author disclosure forms

Video 1 The "Triple-Double" technique of arthroscopic Hill-Sachs remplissage is demonstrated in a left shoulder. The rotator cuff and capsule are sutured into a large Hill-Sachs lesion using three interconnected suture anchors placed into the defect.
